# Pityriasis rubra pilaris with rickets: a rare clinical image

**DOI:** 10.11604/pamj.2022.42.285.36169

**Published:** 2022-08-16

**Authors:** Sangita Shende, Ranjana Sharma

**Affiliations:** 1 Florence Nightingale Training College of Nursing, Datta Meghe Institute of Medical Science University of Delhi (DU), Sawangi Meghe, Wardha, Maharashtra, India,; 2 Department of Medical-Surgical Nursing, Srimati Radhikabai Meghe Memorial College of Nursing, Datta Meghe Institute of Medical Sciences, Sawangi, Wardha, Maharashtra, India

**Keywords:** Pityriasis rubra pilaris, systemic therapy, papulosquamous, Vitamin D, deficiency

## Image in medicine

Pityriasis rubra pilaris (PRP) manifests as well-defined erythematous scaly plaques with follicular keratosis mainly over the elbows and knees. We present a case of a 15-year-old boy who comes to the dermatology department with complaints of itching, dryness, burning sensation, photosensitivity, erythematous skin with exfoliation over face and neck, hyperpigmentation over bilateral hand and trunk fissuring over bilateral feet, ectropion of bilateral lower lids from 3 months. On physical examination, the patient face had lesions and redness on the eyebrow. Skin biopsy and blood investigation revealed the received single, irregular, whitish tissue piece with skin and hair attached measuring 0.2x0.2 cm section shows histopathological features suggestive of pityriasis rubra pilaris. Ortho review call and review of investigations and X-rays advised the patient diagnosed with rickets. The patient was referred to the dermatology department for further management.

**Figure 1:**
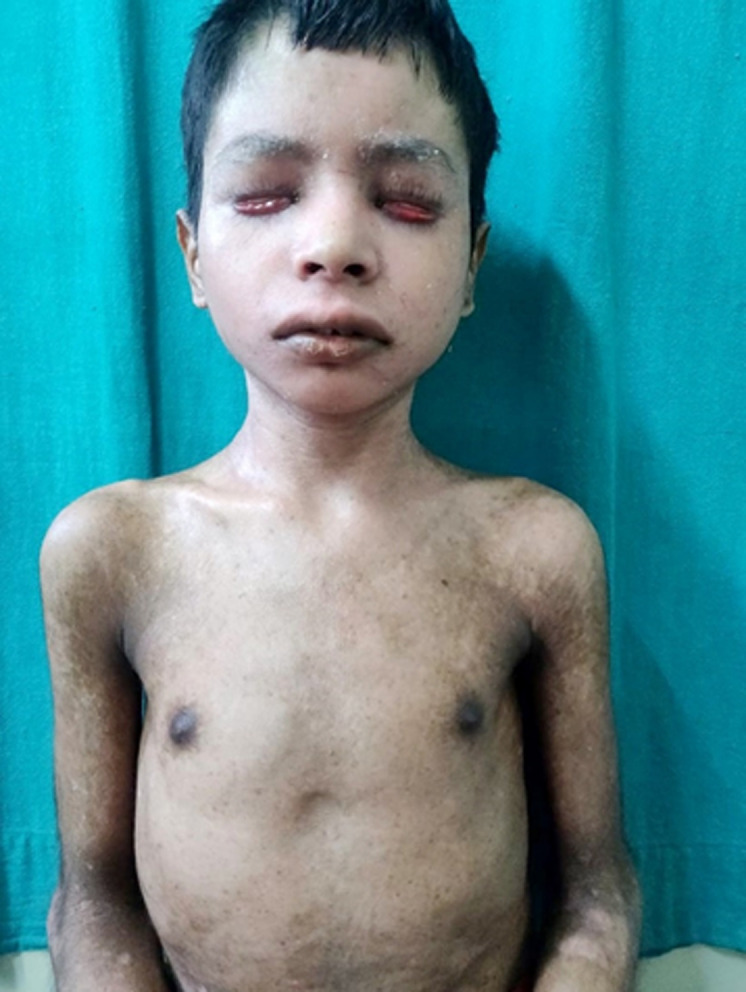
erythematous skin with exfoliation over face and neck, and hyperpigmentation over bilateral hand

